# Transactivation of EGFR by LPS Induces COX-2 Expression in Enterocytes

**DOI:** 10.1371/journal.pone.0038373

**Published:** 2012-05-31

**Authors:** Steven J. McElroy, Stuart Hobbs, Michael Kallen, Noemi Tejera, Michael J. Rosen, Anatoly Grishin, Poojitha Matta, Claus Schneider, Jeffrey Upperman, Henri Ford, D. Brent Polk, Jörn-Hendrik Weitkamp

**Affiliations:** 1 Department of Pediatrics, Vanderbilt University Medical Center, Nashville, Tennessee, United States of America; 2 Department of Cell and Developmental Biology, Vanderbilt University Medical Center, Nashville, Tennessee, United States of America; 3 Vanderbilt University School of Medicine, Nashville, Tennessee, United States of America; 4 Department of Pharmacology and Vanderbilt Institute of Chemical Biology, Vanderbilt University School of Medicine, Nashville, Tennessee, United States of America; 5 Division of Pediatric Surgery, Saban Research Institute, Children's Hospital Los Angeles, Los Angeles, California, United States of America; 6 Departments of Pediatrics and Biochemistry and Molecular Biology, University of Southern California and Children's Hospital Los Angeles, Los Angeles, California, United States of America; University of York, United Kingdom

## Abstract

Necrotizing enterocolitis (NEC) is the leading cause of gastrointestinal morbidity and mortality in preterm infants. NEC is characterized by an exaggerated inflammatory response to bacterial flora leading to bowel necrosis. Bacterial lipopolysaccharide (LPS) mediates inflammation through TLR4 activation and is a key molecule in the pathogenesis of NEC. However, LPS also induces cyclooxygenase-2 (COX-2), which promotes intestinal barrier restitution through stimulation of intestinal cell survival, proliferation, and migration. Epidermal growth factor receptor (EGFR) activation prevents experimental NEC and may play a critical role in LPS-stimulated COX-2 production. We hypothesized that EGFR is required for LPS induction of COX-2 expression. Our data show that inhibiting EGFR kinase activity blocks LPS-induced COX-2 expression in small intestinal epithelial cells. LPS induction of COX-2 requires Src-family kinase signaling while LPS transactivation of EGFR requires matrix metalloprotease (MMP) activity. EGFR tyrosine kinase inhibitors block LPS stimulation of mitogen-activated protein kinase ERK, suggesting an important role of the MAPK/ERK pathway in EGFR-mediated COX-2 expression. LPS stimulates proliferation of IEC-6 cells, but this stimulation is inhibited with either the EGFR kinase inhibitor AG1478, or the selective COX-2 inhibitor Celecoxib. Taken together, these data show that EGFR plays an important role in LPS-induction of COX-2 expression in enterocytes, which may be one mechanism for EGF in inhibition of NEC.

## Introduction

Necrotizing enterocolitis (NEC) is the leading gastrointestinal medical and surgical emergency in premature infants and is the cause of significant mortality and morbidity in this vulnerable population [1]–[3]. NEC is characterized by invasion of the intestine by bacteria followed by an acute, hyper-reactive inflammatory cascade, which leads to loss of epithelial integrity and subsequent bowel necrosis. This process results in up to a 30% mortality rate with severe gastrointestinal and developmental morbidity in survivors [4].

Lipopolysaccharide (LPS) induced activation of the innate immunity pattern recognition molecule Toll-like receptor 4 (TLR4) is associated with increased incidence of NEC in both mice and humans [5], [6]. LPS is a large lipid and polysaccharide structure that is the major component of the outer cell wall of Gram-negative bacteria and acts as an endotoxin. In addition to activating TLR4, LPS has been shown to induce the production of cyclooxygenase-2 (COX-2) in small intestinal epithelial cells [7]. COX-2 is a rate-limiting enzyme in the synthesis of prostanoids from their precursor, arachidonic acid. Elevated COX-2 levels have been demonstrated in both human NEC and animal models of the disease [7], [8]. However, the exact role of COX-2 in NEC pathogenesis remains unclear to date. COX inhibitors such as nonsteroidal anti-inflammatory drugs and glucocorticoids have been linked to neonatal bowel injury [9], and suppression of COX-2 with selective COX-2 inhibitors causes exacerbation of experimental NEC and results in bowel perforation [7], [10]. These findings imply a potential protective role of COX-2 in the intestinal epithelial cells and point to an important role of COX-2 in the reparative response to intestinal injury [11], [12].

Epidermal growth factor (EGF) is critical for the maturation of the fetal and neonatal gastrointestinal tract [13]. EGF is expressed in high concentrations in amniotic fluid, saliva, and breast milk [13], and has been shown to decrease the incidence of NEC in animal models of the disease [14]. EGF signals primarily through the EGF receptor (EGFR), which is a transmembrane glycoprotein with intrinsic tyrosine kinase activity. EGFR is expressed on enterocytes where it induces repair mechanisms following gastrointestinal mucosal injury, promotes cell survival, reduces intestinal inflammation and protects against experimental NEC [14]–[18]. In addition to direct activation by EGF, EGFR can be transactivated indirectly by various extracellular stimuli, including LPS [19]. These transactivation events are important in intestinal epithelial barrier maintenance and can protect the epithelium from apoptosis [20]. Since both LPS and COX-2 are associated with NEC, we sought to test the hypothesis that LPS-mediated COX-2 expression requires EGFR transactivation. Improved insight in the mechanisms regulating enterocyte EGFR and COX-2 signaling is critical for a better understanding of NEC pathogenesis and for developing new targets for therapeutic interventions.

**Figure 1:**
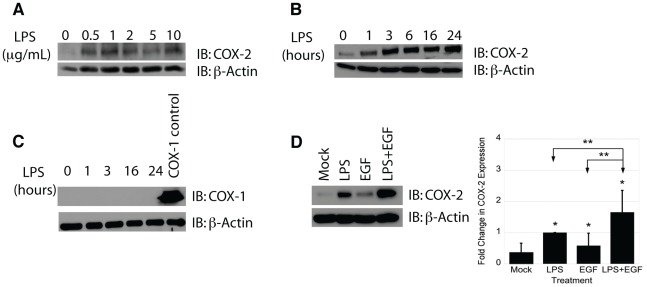
LPS induces COX-2 in IEC-6 cells. A) IEC-6 cells were treated with the indicated concentrations of LPS for 24 hours. B) IEC-6 cells were treated with 2 µg/mL LPS for the indicated time. C) IEC-6 cells were treated with LPS (2 µg/mL) for the indicated times. D) Cells were treated with LPS (2 µg/mL), EGF (10 ng/mL), or co-treated with LPS and EGF for 24 hours. Protein expression was determined by Western blot analysis and densitometry. Treatment with LPS did not induce COX-1 expression in IEC-6 cells. In contrast, treatments with LPS, EGF, or both significantly increased COX-2 expression compared to control (p<0.001,  = 0.05, and <0.001 respectively). Cells treated with LPS and EGF had significantly greater COX-2 expression than by either EGF (p = 0.002) or LPS (p = 0.006) alone. Single asterisks indicate significant differences from control. Double asterisks indicate significant differences between two bracketed conditions.

**Figure 2:**
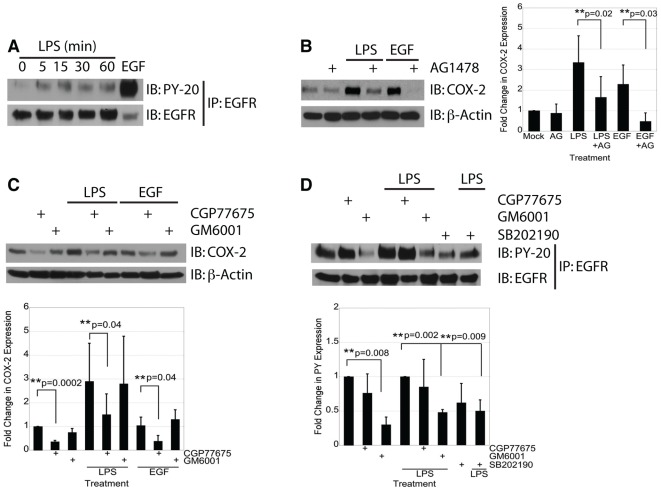
Transactivation of EGFR by LPS induces COX-2 expression in IEC-6 cells in an MMP- and p38-dependent fashion. A) IEC-6 cells were treated with LPS (2 µg/mL) for the indicated time or with EGF (10 ng/mL) for 5 minutes. EGFR immunoprecipitates were assayed for P-EGFR by Western blot analysis. B) Western blot analysis of cells treated with LPS (2 µg/mL) or EGF (10 ng/mL) for 24 hours in the presence or absence of the EGFR kinase inhibitor AG1478 (1 µM). C) IEC-6 cells were stimulated with LPS (2 µg/mL) for 24 hours in the presence or absence of the Src family kinase inhibitor CGP77675 (2 µM) or the MMP inhibitor GM6001 (50 µM). D) IEC-6 cells were stimulated with LPS (2 µg/mL) for 15 minutes in the presence or absence of CGP77675 (2 µM), GM6001 (50 µM), or p38 MAPK inhibitor SB202190 (10 µM). Single asterisks indicate significant differences from control. Double asterisks indicate significant differences between two bracketed conditions.

**Figure 3:**
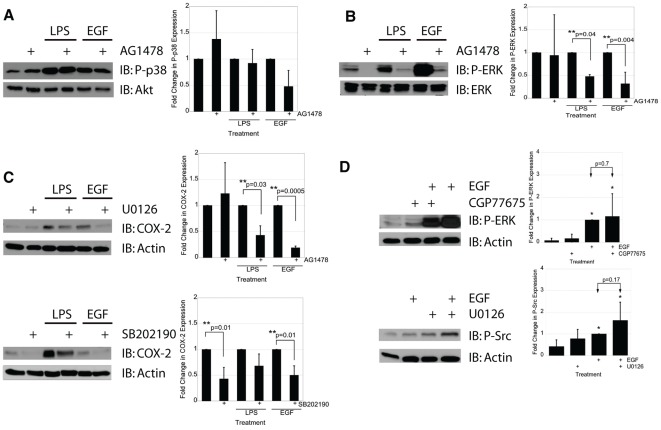
ERK and Src, but not p38, are required for EGFR-mediated induction of COX-2. A) IEC-6 cells were treated with LPS (2 µg/mL) for 15 minutes or with EGF (10 ng/mL) for 5 minutes in the presence or absence of the EGFR kinase inhibitor AG1478 (1 µM). Western blot analysis of P-p38 MAPK showed no significant difference in p38 activation in the presence of EGFR inhibition. B) IEC-6 cells stimulated with LPS (2 µg/mL) for 15 minutes or with EGF (10 ng/mL) for 5 minutes in the presence or absence of AG1478 (1 µM). C) IEC-6 cells were treated with LPS (2 µg/mL) for 24 hours or with EGF (10 ng/mL) for 5 minutes in the presence or absence of the ERK1/2 inhibitor U0126 (10 µM) or the p38 inhibitor SB202190 (10 µM) as shown, and COX-2 expression was determined using Western blot analysis. D) IEC-6 cells were treated with EGF (10 ng/mL) for 5 minutes in the presence or absence of the Src family kinase inhibitor CGP77675 (2 µM) and analyzed for P-ERK activation using Western blot analysis. Src inhibition had no effect on EGF-induced P-ERK (p = 0.7). IEC-6 cells were also treated with EGF (10 ng/mL) for 5 minutes in the presence or absence of the ERK kinase inhibitor U1026 (10 µM) and analyzed for P-Src activation using Western blot analysis. ERK inhibition had no effect on EGF-induced P-Src activation (p = 0.17). Single asterisks indicate significant differences from control. Double asterisks indicate significant differences between two bracketed conditions.

**Figure 4:**
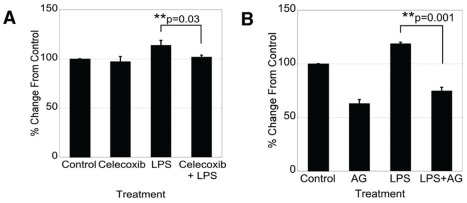
Stimulation of IEC-6 cell proliferation by LPS requires COX-2 activity. IEC-6 cells were treated with LPS (2 µg/mL) for 48 hours in the presence or absence of A) Celecoxib (10 µM) or B) AG1478 (1 µM). Cell numbers were determined by a Nucleocassette counter. Celecoxib and AG1478 treatment significantly blocked LPS-induced proliferation (p = 0.03 and 0.001 respectively).

**Figure 5:**
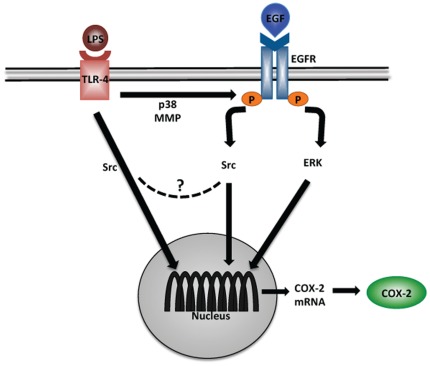
Proposed mechanism of LPS-induced COX-2 stimulation via EGFR transactivation. LPS can induce COX-2 production either directly through TLR-4 signaling or via EGFR transactivation. EGFR transactivation requires p38 and MMP activity. Following EGFR transactivation, COX-2 production can be stimulated either through ERK- or Src-mediated pathways. Although our data suggest that LPS and EGF can induce COX-2 expression through separate pathways, we cannot exclude that the pathway merges downstream prior to transcription.

## Materials and Methods

### Cell culture

IEC-6 cells (ATCC, Manassas, VA) were grown as a monolayer in DMEM media supplemented with 5% FBS (Hyclone), 0.1% Insulin-Transferrin-Selenium (ITS) and 1% Penicillin/Streptomycin (BD Biosciences, San Jose, CA), in a humidified atmosphere containing 5% CO_2_. Prior to experiments, cells were cultured for 24 hours and then serum starved in DMEM media without FBS for 16 hours. Unless otherwise noted, the cells were then treated with 2 µg/mL LPS from *Escherichia coli* O127:B8 (Sigma, St. Louis, MO) or 10 ng/mL murine EGF (Peprotech, Rocky Hill, NJ) for the indicated durations. In pharmacologic studies, IEC-6 cells were pre-treated with inhibitors for 1 hour before further stimulation.

### Antibodies and inhibitors

Western blot antibodies used for these experiments included: beta-Actin (Sigma, St. Louis, MO), COX-1 and COX-2 (Cayman Chemical, Ann Arbor, MI), anti-rabbit and anti-mouse horseradish peroxidase-conjugated secondary antibodies, phospho-Y845-EGFR, phospho-ERK1/2, ERK1/2, and phospho-p38 (Cell Signaling Technology, Beverly, MA). Anti-EGFR antibodies were purchased from Millipore (Bedford, MA) and HRP-conjugated anti-phospho-tyrosine antibody was purchased from BD Biosciences (Franklin Lakes, NJ). Specific inhibitors were from the following sources: EGFR kinase inhibitor AG1478, MMP inhibitor GM6001, p38 MAPK inhibitor SB202190 were purchased from EMD Chemicals (Gibbstown, NJ); the ERK1/2 inhibitor U0126 was purchased from Cell Signaling Technology (Boston, MA); and the selective COX-2 inhibitor Celecoxib was purchased from Sigma (St. Louis, MO).

### Cell lysates and Western blotting

Cell monolayers were washed twice with ice cold PBS and scraped on ice into cold lysis buffer (1% Triton X-100, 10% 150 mM NaCl, 50 mM Tris, pH 7.4). Cellular lysates were cleared and boiled in Laemmli sample buffer [30]. Protein expression and phosphorylation was determined by Western blot analysis.

### Immunoprecipitation

Cellular lysates were pre-cleared by incubating with protein AG agarose beads (Santa Cruz Biotechnology, Santa Cruz, CA) for 30 min followed by centrifugation. Supernatants were incubated with 2 µg anti-EGFR antibody for 1 hour at 4°C, and then for 1 hour at 4°C with protein AG agarose beads. Immunocomplexes were collected by centrifugation, washed three times in lysis buffer, and boiled in Laemmli sample buffer for SDS-PAGE Western blot analysis.

### Cell proliferation assays

1.75×10^4^ IEC-6 cells/well were seeded in 24-well plate wells and grown for 24 hours in DMEM media (supplemented with FBS and ITS) followed by serum starvation for 16 hours. The cell monolayers were pretreated with inhibitors for 1 hour followed by treatment with or without LPS for 48 hours. Cells were counted using a Nucleocounter (New Brunswick, Edison, NJ) using manufacturers protocols.

### Replicates and statistical analysis

All data are representative of at least three independent experiments. Statistical significance of differences between means from two groups was assessed with a Student's t-test analysis. When comparisons were made amongst three or more groups, analysis of variance was applied first as a global test for differences. Pre-determined pair-wise comparisons were then made using Student's t-test only when an overall effect was detected through analysis of variance. Minimum level of significance was set at 0.05.

## Results

### LPS and EGF induce COX-2 protein expression in IEC-6 cells

To determine the optimal conditions for LPS stimulation of COX-2, IEC-6 cells were treated with varying concentrations of LPS (Fig. 1A) and for various time periods (Fig. 1B) as shown. We chose the 24-hour time-point for our studies, because the level of COX-2 protein induction was the strongest and most reproducible at that time by Western blot. Twenty-four hour treatments with LPS induced COX-2 expression at all concentrations, but the induction was most consistent at 2 µg/mL, which was the LPS dose chosen for the remainder of the experiments unless otherwise noted. At 2 µg/mL LPS induced COX-2 beginning at 1 hour of treatment and persisting for at least 24 hours (Fig. 1B). To determine if LPS specifically induced COX-2, we examined LPS-treated IEC-6 cells for COX-1 induction. LPS caused no induction of COX-1 at any time point (Fig. 1C). Since both LPS and EGF have been shown to stimulate COX-2, we next attempted to determine if the effects of LPS and EGF were additive. IEC-6 cells were treated with LPS, EGF, or both and compared to controls (Fig. 1D). Treatment of IEC-6 cells with either LPS or EGF significantly increased COX-2 expression over controls (p<0.001 for LPS and p = 0.05 for EGF). However, combined LPS and EGF treatments significantly increased COX-2 expression over controls (p<0.001) and over both LPS (p = 0.006) and EGF (p = 0.002) treatments alone. This suggests that LPS and EGF can induce COX-2 through separate pathways, although we cannot exclude a converging pathway downstream.

### Transactivation of EGFR by LPS induces COX-2 expression in IEC-6 cells

LPS and EGF can both stimulate COX-2 expression directly, however, LPS has also been shown to transactivate EGFR in intestinal epithelial cells [19]. To understand the potential role of EGFR transactivation in LPS stimulation of COX-2 we first examined the ability of LPS to directly stimulate EGFR phosphorylation. In IEC-6 cells treated with LPS for varying times, increased phosphorylation of EGFR was observed after 5–60 minutes of treatment (Fig. 2A). Since LPS induces both COX-2 expression and EGFR phosphorylation, and activation of EGFR can induce COX-2, we wanted to determine if EGFR was required for LPS-induced activation of COX-2. To examine the requirement of EGFR, IEC-6 cells were pretreated with AG1478, a small molecule inhibitor of EGFR kinase activity, prior to treatment with LPS or EGF. Pretreatment of IEC-6 cells with AG1478 significantly reduced both LPS- and EGF-induced COX-2 expression, indicating that EGFR is important for both LPS and EGF induction pathways of COX-2 (p = 0.02 and 0.03, respectively) (Fig. 2B).

### Transactivation of EGFR by LPS requires MMP and p38 activity

Our data show that transactivation of EGFR by LPS plays a role in COX-2 induction. To further understand the mechanism of this pathway, we next examined the role of two known signaling pathways involved in EGFR transactivation, Src family kinases and matrix metalloproteases, in activation of COX-2 by LPS and EGF. IEC-6 cells were pretreated with CGP77675 (a specific Src family kinase inhibitor) or GM6001 (a general MMP inhibitor), prior to stimulation with either LPS or EGF. COX-2 protein expression was measured by Western blot analysis (Fig. 2C). The Src family inhibitor CGP77675 significantly decreased basal, LPS-induced, and EGF-induced expression of COX-2 (p = 0.002, 0.04, and 0.04 respectively). By contrast, the MMP inhibitor GM6001 had no effect on either basal or induced COX-2 expression. These data support the hypothesis that LPS induces COX-2 through a Src-dependent mechanism. To determine if Src family members, or MMPs play a role in the activation of EGFR by LPS, IEC-6 cells were pretreated with CGP77675 or GM6001 prior to stimulation with LPS. GM6001 blocked baseline and LPS-stimulated activation of EGFR (p = 0.008 and 0.002 respectively) (Fig. 2D), suggesting that MMPs are required for activation of EGFR by LPS. While the Src family inhibitor CGP77675 blocked EGF-stimulated activation of COX-2, it had no effect on LPS-induced phosphorylation of EGFR, suggesting that Src is involved downstream of EGFR transactivation. We additionally examined the role of the MAPK p38 in LPS activation of EGFR, which previously has been reported to play a role in LPS induced expression of COX-2 [7], [21]. IEC-6 cells were pretreated with the p38 inhibitor SB202190 prior to treatment with LPS. Inhibition of p38 significantly blocked transactivation of EGFR by LPS (p = 0.009) (Fig. 2D). Taken together, these data suggest that LPS-induced transactivation of EGFR requires the activity of MMPs and p38, whereas LPS- and EGF-induced expression of COX-2 require the activity of Src family kinases.

### LPS couples to EGFR downstream activation of MAPK ERK

Grishin *et al* previously reported that LPS induces COX-2 via a noncanonical p38 MAPK pathway in IEC-6 cells [7], [21]. Our data show that p38 is necessary for activation of EGFR by LPS (Fig. 2D). Therefore, we tested whether transactivation of EGFR by LPS is coupled to p38 MAPK activation. IEC-6 cells were pretreated with AG1478 followed by stimulation with LPS or EGF. While p38 was required for LPS activation of EGFR, blocking EGFR kinase activity did not inhibit p38 activation by either LPS or EGF (Fig. 3A), suggesting that p38 is involved in a pathway of EGFR transactivation that does not depend on EGFR kinase activity. To gain insight into the mechanism of COX-2 induction via EGFR we analyzed the ERK/MAPK, which is another downstream signaling target of EGFR. IEC-6 cells were pretreated with AG1478 followed by treatment with LPS or EGF, and ERK activation was examined with Western blot analysis (Fig. 3B). Both LPS and EGF were stimulated ERK activation. Blocking the EGFR kinase activity with AG1478 significantly reduced both LPS- and EGF-induction of ERK (p = 0.04 and 0.004 respectively) implicating ERK as a signaling target of activated EGFR. To determine whether ERK activity plays a role in LPS-induced expression of COX-2, we pretreated cells with U0126, an inhibitor of the ERK signaling pathway prior to treatment with either LPS or EGF (Fig. 3C). Blocking ERK activation significantly inhibited both LPS- and EGF- induced expression of COX-2 (p = 0.03 and 0.0005 respectively), indicating an important role of the ERK pathway in COX-2-induction downstream of LPS-transactivated EGFR. We repeated this experiment using the p38 inhibitor SB202190 to determine if p38 inhibition would have similar effects. Blocking p38 activity significantly inhibited baseline and EGF-induced but not LPS-stimulated expression of COX-2 (p = 0.01).

Since our data indicated that both ERK and Src are required for EGFR activation of COX-2, we next sought to determine if these two kinases act in the same pathway or in distinct pathways. IEC-6 cells were pre-treated with either the Src inhibitor CGP77675 or the ERK inhibitor U0126 prior to treatment with EGF. Cells were examined for phosphorylation of ERK and Src by Western blot analysis (Fig. 3D). Neither inhibitor was able to quench the downstream activation of the intended target implying that Src and ERK belong to different downstream pathways of EGFR-induced COX-2 expression.

### COX-2 and EGFR inhibition both block LPS-stimulated cell proliferation

LPS-induced COX-2 expression has been proposed to play a role in enterocyte proliferation [22], [23]. Therefore, we tested the hypothesis that disruption of LPS induction of COX-2 both directly and through disruption of EGFR transactivation would reduce IEC-6 cell growth. Treatment of IEC-6 cells with LPS stimulated proliferation, which is consistent with the mitogenic effects of EGFR and ERK (Fig. 4A and 4B). Both the selective COX-2 inhibitor Celecoxib (Fig. 4A) and the selective EGFR inhibitor AG1478 (Fig. 4B) were able to significantly decrease LPS-induced proliferation (p = 0.03 and 0.001 respectively). These data further demonstrate an overlap in the biological activities of EGFR and COX-2, and suggest that COX-2 and EGFR play critical roles in the mitogenic response to LPS.

## Discussion

This study delineates an important pathway of LPS-induced COX-2 upregulation through EGFR transactivation, suggesting a novel role of EGFR in enterocyte homeostasis and, potentially, in the protection against NEC. Although the pathogenesis of NEC continues to be explored, the unifying hypothesis includes mucosal injury of the small intestine, followed by bacterial translocation and an exaggerated inflammatory response to endotoxin (LPS) [24]. LPS binds Toll-like receptor 4 (TLR4), leading to activation of nuclear factor (NF)-κB and subsequent proinflammatory cytokine release by enterocytes and other cells [25]. Despite this proinflammatory mechanism, the role of LPS in inflammatory disorders of the intestine is not entirely clear. Whereas LPS has been shown to reduce enterocyte migration and proliferation via TLR4, which may impair intestinal healing [26], [27], LPS-induced COX-2 expression stimulates proliferation of colonocytes and repair of colonic epithelium [22]. Regarding the pathophysiology of NEC, several reports have demonstrated the role of TLR4 as causative for the disease [6], [28], and yet Grishin and colleagues reported LPS stimulation of COX-2 was protective in experimental NEC [7]. Our data help to explain this dichotomy by showing that LPS can induce COX-2 expression in enterocytes through EGFR transactivation. Although our data suggests that LPS and EGF can induce COX-2 through separate pathways, we cannot exclude a converging pathway downstream.

One goal of this study was to identify signaling pathways that mediate COX-2 induction after LPS transactivation of EGFR. By using specific inhibitors of EGFR kinase activity we established that induction of COX-2 by LPS involves EGFR transactivation. Our data also demonstrate that transactivation of EGFR by LPS is dependent on p38 and MMP activity. Once EGFR is transactivated, further induction of COX-2 depends on activation of ERK and Src, two downstream targets of EGFR. Whereas both ERK and Src are required for induction of COX-2 by LPS via transactivation of EGFR, they appear to act in distinct parallel pathways (Fig. 5).

We speculate that MMPs are involved in transactivation of EGFR by LPS, but none of these phosphotyrosines on EGFR couple to COX-2 induction. In this case Src may actually be involved in transactivation of EGFR phosphotyrosines that do couple to COX-2 activation, but these phosphotyrosines are not visible to the phosphotyrosine antibody that we used in this study.

Grishin and colleagues previously reported an essential role of the p38 MAPK in COX-2 upregulation in enterocytes [7], [21]. Our data show that p38 is required for LPS induced transactivation of EGFR, but not for subsequent EGFR-mediated COX-2 induction. Thus, inflammatory upregulation of COX-2 in the intestine is more complex than previously thought and may involve p38-dependent and p38-independent pathways.

Our central finding is the critical involvement of EGFR in LPS-induced COX-2 expression in enterocytes. EGFR is abundantly expressed in premature intestinal epithelial cells [29] and EGFR and its ligands have long been recognized as protective factors in NEC [18], [30]–[32]. The significance of MAPK-independent EGFR signaling in LPS-induced wound repair has been previously described in airway epithelial cells [33]. Interestingly, the effects of LPS on wound healing were dose-dependent [33], which is yet another illustration of the delicate balance between injury and repair that characterizes the complex molecular pathways in epithelium-microbial interactions. The effects of LPS and EGF on EGFR signaling may also be time-dependent. While prolonged receptor stimulation with LPS did not increase COX-2 expression compared to 24 hour pretreatment alone, sustained stimulation for 6 hours with EGF enhanced the COX-2 signal by Western blot (data not shown).

Inhibition of LPS-stimulated enterocyte proliferation by the selective COX-2 inhibitor Celecoxib, and by the selective EGFR kinase inhibitor AG1478 implies a possible role of EGFR-mediated induction of COX-2 in epithelial restitution. Based on our data, we propose a role of EGFR transactivation by LPS in the inflammatory upregulation of COX-2 in the intestine.
